# Anti-arthritic property of crude extracts of *Piptadeniastrum africanum* (Mimosaceae) in complete Freund’s adjuvant-induced arthritis in rats

**DOI:** 10.1186/s12906-017-1623-5

**Published:** 2017-02-15

**Authors:** Marius Mbiantcha, Jabeen Almas, Simjee U. Shabana, Dastagir Nida, Faheem Aisha

**Affiliations:** 1 Laboratory of Animal Physiology and Phytopharmacology, Faculty of Science, University of Dschang, P.O. Box 67, Dschang, Cameroon; 2 Dr. Panjwani Center for Molecular Medicine and Drug Research, International Center for Chemical and Biological Sciences, University of Karachi, Karachi, 75270 Pakistan; 3 H.E.J. Research Institute of Chemistry, International Center for Chemical and Biological Sciences, University of Karachi, Karachi, 75270 Pakistan

**Keywords:** *Piptadeniastrum africanum*, Immunomodulation, Oxidative burst, T-cell proliferation, Cytotoxicity, Rheumatoid arthritis

## Abstract

**Background:**

Rheumatoid arthritis, disease of unknown causes is a rheumatic and autoimmune pathology, recognised for its increasing frequency and its adverse consequences. It is a disease that occurs in most cases between 50 and 60 years and women are more affected than men. This study aimed at evaluating immunomodulatory and anti-arthritis capacity of aqueous and methanol extracts of stem bark of *Piptadeniastrum africanum* (Mimosaceae).

**Methods:**

ROS production from phagocytes, proliferation of T-cells, TNF-α and IL-1β production and cytotoxicity were performed by using chemiluminescence technique, liquid scintillation counter, ELISA and MTT assay, respectively. Anti-arthritic activity was evaluated using a model of adjuvant induced arthritis.

**Results:**

Methanol and aqueous extracts of *Piptadeniastrum africanum* significantly (*P < 0.001*) inhibited extracellular and intracellular ROS production. These extracts also possess significant (*P < 0.001*) inhibitory activity on T-cell proliferation other than reduced TNF-α and IL-1β production. *Piptadeniastrum africanum* also significantly exhibited antiarthritic activity in complete Freund’s adjuvant induced arthritis in rat associated with a significant anti-inflammatory and anti-hyperalgesia activity.

**Conclusions:**

Immunomodulatory, anti-inflammatory, antihyperalgesia and anti-arthritis potential revealed in this study approve that, *Piptadeniastrum africanum* is a plant rich in compounds with anti-arthritic properties.

## Background

Rheumatoid arthritis (RA), that affects between 0.3 and 1% people in the world, is considered as a chronic and debilitating pathology, and globally as a most frequent inflammatory rheumatic disease. The disease appears in most cases between 50 and 60 years of age and women are more affected than men [1, 2]. It affects life and limits the movement of people [3, 4]. In people with RA, a deformation of joints is observed with increased in volume and loss of function, cartilage and bone destruction; these people suffer with a remarkable reduction of daily life quality which can lead to depression [5]. This disease mainly affects the ankle joint resulting in a gradual painful swelling, exaggerated and abnormal development of the synovium, pannus formation and changes in the morphology of the joint. [5, 6] RA progresses in three stages: swelling, warmth, pain and redness around the joint due to the swelling of the synovial membrane; thickening of the synovial membrane due to the division and the rapid growth of cells or pannus; release of enzymes by the inflamed cells that can digest bone and cartilage resulting in loss of shape and alignment of the joint involved with more pain and loss of motion [7].

Immunology holds a dominant role in the understanding of rheumatic diseases [8]. In rheumatoid synovium, we note the presence of monocytes, mast cells, macrophages, polymorphonuclear cells and fibroblast synoviocytes [9, 10]. Activated macrophages and synoviocytes will secrete large amounts of proteases, cytokines IL-1β and TNF-α [11]. These cytokines are strong pro-inflammatory mediators in the cascades of information transmission between inflammatory cells in RA synovitis [12]. IL-1β and TNF-α play a particularly important role by causing the liberation of metalloproteases (collagenases, stromelysin) and prostaglandins (PGE_2_) by activated cells (fibroblastes, macrophages, synovial dendritic cells) [13]. This causes destruction of cartilage tissue, bone resorption and chronic proliferation of synoviocytes cells [14]. IL-1β and TNF-α have synergistic effects in aggravating the inflammation, so by inhibiting TNF-α, we can block or reduce the activity of IL-1β [11]. TNF-α appears as the cytokine of inflammation but is also capable of inducing cartilage lesions, while IL-1β which is also important in inflammation, appears especially as the cytokine responsible of cartilage lesions and delays healing of lesions [15]. TNF-α also induces the release of other cytokines as IL-6 (inducer of the hepatic acute phase response) and IL-8 (chemotactic factor for neutrophils), which puts TNF-α at the head of processes including pro-inflammatory cytokines [15]. The major role of these mediators in the immunopathology of RA has led to the elaboration and the use of targeted therapies anti-TNF-α and anti-IL-1β [14]. Otherwise, in RA, oxidative stress is recognized as an important parameter in pathogenicity with oxygen radicals which are factors involved in tissue destruction [16, 17]. In the inflamed or painful joints, macrophages, chondrocytes and neutrophils can produce the ROS (reactive oxygen species) [18].

The evaluation of a drug that can prevent the cause of arthritis or act during the stages of installation of arthritis requires the use of an evaluation model that produced in animals the same type of arthritis in humans [19]. The evaluation that the different classes of compounds having anti-arthritic activity in clinical use requires the use of appropriate animal models. However, the choice of the model needs not only the morphological similarities to human diseases, but also the ability of the model to predict the effectiveness of treatment in humans [19, 20]. The pathophysiological study, pharmacological control of inflammatory processes, analgesic, anti-inflammatory and/or antiarthritis effects of drugs can be evaluated after injection of complete freund’s adjuvant on rat [21], it is also widely used in the evaluation of systemic changes observed in chronic inflammation [22], using the CFA model induced arthritis on rat in experimental research is justified by the fact that therapeutic compounds active in this model are also active for the vast majority of humans with RA [23].

Based on the fact that, human civilization uses natural products with therapeutic properties for many decades, it is obvious that, medicinal plants offer a real and permanent solution to many health problems. Today, the modulation of the immune response by various natural products as well as by compounds obtained through chemical synthesis have already been demonstrated by many researchers [24, 25]. Inflammatory mediators, including cytokines and free radicals play a vital role in many diseases, putting inflammation and oxidative stress at the center of many pathological conditions, among the most frequent health problems in the world which include the dysfunction of the immune system (RA, atherosclerosis, inflammatory disease, diabetes and neurodegenerative disorders) [26]. It is therefore necessary to find new therapeutic approaches that target the inflammatory parameters mainly involved in the pathogenesis of the disease [27].

*Piptadeniastrum africanum* (*P. africanum*) belong to the family Mimosaceae. In Cameroon, it is used for the management of constipation, anaemia, lumbago, meningitis, pains, edema, rheumatism, convulsion, gastric ulcer and wound treatment [28–30]. Several plant extracts belonging to the Mimosaceae family showed anti-arthritic properties. These include *Acacia auriculiformis* [26], *Acacia polyantha* [31] and *Albizia lebbeck* [32]. Previous works have shown that, *P. africanum* possesses antibacterial properties and has no toxicological effects under the threshold of 5 mg/kg in rat [33, 34]. Previous studies have also reported that, the stem bark of *P. africanum* given orally to rats, possesses gastro-protective and ulcer healing properties [35], antiproliferative [36] and antinociceptive activities, as well as anti-inflammatory properties of the model of chronic inflammation induced by formalin [37]. Recent phytochemical studies on the root of *P. africanum* have revealed the presence of flavonoids, alkaloids, steroids, triterpenoids and saponins [36, 38]. Several of these chemical compounds have already shown their interesting effect both in immunomodulation and in the treatment of rheumatoid arthritis induced by CFA [39, 40].

CFA induced RA model on rat is a chronic inflammation including multiple systemic change with synovial hyperplasia resulting from a great proliferative cellular infiltrate of leukocytes and an abnormal increase in levels of many cytokines (particularly IL-1β and TNF-α), significant release of ROS and consequent cartilage and bone destruction with swelling, deformation and loss of function of joint. [18, 41] Based on the fact that, the stem bark of *P. africanum* possess anti-inflammatory properties on chronic inflammatory model, and the fact that preliminary in vitro assay have shown the suppressive properties of the production of pro inflammatory cytokines, antiproliferative properties and inhibiting ROS production, we chose in our study in vivo, to determine the anti-arthritic properties of this plant using the model of arthritis induced by CFA on rat.

In this study, we firstly describe in vivo, anti-inflammatory, antihyperalgesia and antiarthritis effects of stem bark of *P. africanum* (aqueous and methanol extracts) and its significant effect on systemic changes, and secondly, in vitro studies to evaluate immunomodulatory, antiproliferative and cytotoxic effects. Both extracts were analysed for their inhibitory property on intra and extracellular ROS as well as cytokines production.

## Methods

### Reagents, chemicals, and equipments

Lymphocytes Separation Medium, Luminol, Indomethacin as Hanks Balance Salts Solution and Lucigenin were obtained from MP Biomedicals Inc., Research Organics and Sigma ; Dimethylsulphoxide, ethanol and ammonium chloride of analytical grades from Merck Chemicals, Darmstadt, Germany; Zymosan A as Phorbol myristate acetate from Fluka and Human monocytic leukemia cells from European Collection of Cell Cultures; human TNF-α and IL-1β ELISA Kit from R&D systems, Minneapolis, USA and glass fiber filter and cell harvester from INOTECH, Dottikon, Switzerland.

### Experimental animals

In the present study, female *Wistar* rats (150–200 g, 10 to 12 weeks of age) were used. Animal house of H.E.J. Research Institute of Chemistry, International Center for Chemical and Biological Sciences (ICCBS), University of Karachi, Pakistan, provided all the animals. During 1 week acclimatization (22 ± 1 °C temperature and 50–80% humidity), with 12 h cycle variation between the light and dark, freely, animals consumed a standard diet for rodents and water filtered beforehand. The coherence of the effects of administered treatments were determined using a minimum possible number of rats. The treatment of animals was in agreement with the Institutional Animal Care, Use and Standards Committee (IACUC) of ICCBS were followed (Protocol No. 1209004), and the study protocols accepted by the ethics committee of ICCBS were followed, University of Karachi, Pakistan.

### Collection and preparation of plant material

*P. africanum* (Hook. f.), collected from the district of Bokito in Cameroon (Central Region), authenticated by referring to other specimens (N° 12115/SRF) preserved at the Cameroon National Herbarium (Yaounde), was used for this study. After collection of fresh stem bark of *P. africanum*, they were cut into small pieces, dried out of the sun and then crushed into a fine powder.

### Plant material

Aqueous extract: 560 g of *P. africanum* stem bark powder was mixed with distilled water (3 l), the mixture was wormed to boil (20 min, decoction), cooled (30 min) at room temperature followed by filtration (Whatman filter paper no.1) and evaporation (drying oven set at 40 °C) afforded 19.72 g (3.25% yields) of aqueous extract.

Methanol extract was obtained by maceration of 200 g of powder in 1.5 l of methanol for 72 h. Filtration (Whatman filter paper) of the mixture followed the concentration of the filtrate (rotary evaporator, 65 °C) afforded 18.81 g (28.08% yields) of methanol extract.

### In vitro assays

#### Isolation of human polymorpho neutrophils (PMNs)

Ten milliliters of venous blood obtained aseptically from a healthy volunteer donor (31 years of age) were introduced into a tube containing heparin as an anticoagulant. The method of Ficoll hypaque density gradient centrifugation was used to isolated neutrophils [42]. Briefly, on a 45 ml empty centrifuge tube, whole blood, lymphocytes separation medium (LSM) and HBSS were mixed in equal volumes. After 30 min, the supernatant was sampled and slowly introduced into empty centrifuge tubes of 15 ml containing 5 ml of lymphocytes separation medium (LSM) and centrifuged (400 g, 20 min, room temperature). After discarding the supernatant, distilled water (1 ml) was added to the pellet for exactly one minute for the lysis of RBCs and 1 ml of HBSS^−−^(2x) was then added to stop the lysis. Then 5 ml of HBSS was again added to the tubes and the tubes were centrifuged (300 g, 10 min, 4°C), discarded the supernatant and introduced again HBSS^−−^ (1ml) and stored (in ice). Trypan blue exclusion method was used to check the viability and the cells counted by haemocytometer. A cell concentration of 1 × 10^6^ cells/ml was used for tests.

#### Peritoneal macrophages isolation from mice

NMRI mice (18 – 25 g), immunized with FBS (1 ml) injected intraperitoneally using sterile 1 ml syringe, were kept for 72 h in the animal house and killed by cervical dislocation. 10 ml of 10% RPMI medium was injected in the peritoneum cavity after sterilizing whole animal bodies by dipping into 70% ethanol. Peritoneum cavity was then massaged for 2 min and the abdominal skin was cut from the lower side and retracted to expose peritoneum cavity. The injected RPMI containing macrophage was collected with the help of a sterile syringe from peritoneum, then centrifuged for 20 min (400 g, 4°C). After discarding the supernatant, the pellet was washed with incomplete RPMI media, then centrifuged for 10 min (300 g, 4 °C), then added 1 ml of incomplete RPMI media/HBSS. Trypan blue exclusion method was used to check viability and count cell by haemocytometer, a cell concentration of 1 × 10^6^ cells/ml was used for tests [43, 44].

#### Chemiluminescence assay

Chemiluminescence test was done as described previously with modifications [43, 44]. White plates 96 wells were used for testing. 25 μl of plant extracts (3.1 to 100 μl/ml) or ibuprofen were mixed with 25 μl of whole blood (diluted in HBSS^++^ (1:50)) or PMNs (1 × 10^6^) or macrophages (2 × 10^6^) cells, except for control wells that received only the HBSS^++^ and cells without extract. After 20 min incubation of plates at 37°C (thermostated chamber of Luminometer), to reach 100 μl volume/well, 25 μl of Serum Opsonized Zymosan/PMA and/or 25 μl of luminol/lucigenin (7 × 10^5^ M) were added. The results of this test were obtained as Relative light units (RLU) [44]. The following formula was used to calculate inhibition percentage (%) for each compound:

$$ \mathrm{Inhibition}\ \left(\%\right) = \frac{\left({\mathrm{RLU}}_{\mathrm{control}}\hbox{--}\ {\mathrm{RLU}}_{\mathrm{sample}}\right) \times 100\ \%}{{\mathrm{RLU}}_{\mathrm{control}}} $$

#### Cytokine assay

To maintain THP-1 (Human monocytic leukemia cells), endotoxin free RPMI1640 was used. The cultivated cells (2.5 × 10^5^ cells/ml) in 75cc flasks up to 70% confluence were introduced into 24 wells tissue culture plates. PMA was used for differentiated cells into macrophage like cells (20 ng/ml) and incubated for 24 h at 37 °C in 5% CO_2_. *E. coli* Lipopolysacchride B was used to stimulate cells (50 ng/ml), treated with extracts (50, 10 and 2 μg/ml), plate were incubated (4 h, 37 °C) in CO_2_ (5%). Finally the supernatants was collected to analyze the level cytokines using human TNF-α and IL-1β ELISA Kit [45].

#### T-Cell proliferation assay [41]

Briefly, in white 96 wells round bottom plates, 50 μl of extract (2, 10 and 50 μg/ml) or prednisolone diluted in 5% RPMI were introduced. Each dilution were triplicated, then 50 μl of isolated T-cells (2 × 10^6^ cells/ml) were added and stimulated with 50 μl of phytohemagglutinin-L (PHAL) (7.5 μg/ml). Negative control wells received only cells (50 μl) and 5% RPMI (150 μl) and positive control received cells (50 μl), PHA (50 μl) and 5% RPMI (100 μl). After the incubation of plates (72 h, 37 °C) in CO_2_ (5%) incubator, 25 μl of 0.5 μCi/well (methyl 7 3H) thymidine were used to pulse the cultures, then plates were further incubated (18 h) and glass fiber filter was used to harvest the cells. LS65000 liquid scintillation counter was used to determine the level of the thymidine integrated into the cells. Percent inhibition was determined by using the counts per minute (CPM) of each well according to the formula:

$$ \mathrm{Inhibitory}\ \mathrm{activity}\ \left(\%\right) = \frac{{\mathrm{CPM}}_{\left(\mathrm{Control}\ \mathrm{group}\right)}\hbox{--}\ {\mathrm{CPM}}_{\left(\mathrm{Experiment}\ \mathrm{group}\right)}}{{\mathrm{CPM}}_{\left(\mathrm{Control}\ \mathrm{group}\right)}}\times 100 $$

#### MTT Cytotoxicity assay

In vitro cytotoxicity of extracts was evaluated by MTT assay described by Scholz et al. [46]. 96 welled flat bottom plates containing 100 μl of cell suspension (6 × 10^4^ cells/ml) were incubated (24 h, 37 °C) in CO_2_ (5%). After removing the media in each well, extracts (3.1–100 μg/ml) and complete DMEM were added (200 μl final volume). Positive control wells contained cells (100 μl) and complete DMEM and in negative control 0.5% triton X100 (2 μl) were add. After incubation (48 h, 37 °C) in CO_2_ incubator, the supernatant was removed, MTT (50 μl, 0.5 mg/ml) diluted in PBS (5 mg/ml) added and plates were incubated again (4 h). MTT was carefully aspirated and DMSO (100 μl) was added with agitation (10–15 min) in an orbital shaker. The spectrophotometer was used at 540 nm for absorbance. The percent inhibition or decrease in cells viability was obtained the formula:

$$ \%\mathrm{Inhibition} = 100\ \hbox{--} \frac{{\mathrm{OD}}_{\mathrm{test}\ \mathrm{group}}-{\mathrm{OD}}_{\mathrm{blank}}}{{\mathrm{OD}}_{\mathrm{Control}\ \mathrm{group}} - {\mathrm{OD}}_{\mathrm{blank}}} \times 100 $$

### In vivo assays

#### Induction of AIA (adjuvant induced arthritis)

To induce arthritis, animals were first anesthetized with a small amount of ether vapor, then 100 μl of freshly prepared CFA (10 mg/ml) dissolved in mineral oil (sterile) was injected delicately into the hindpaw [47]. Normal animals received 100 μl of sterile mineral oil.

#### Treatment regimen

After classifying and grouping animals according to their weight, each animal was marked by the tail with a number and placed in a cage with a letter identifying the cage. By this process, 42 female rats were distributed into 7 groups (6 rats each). Group 1 (healthy control) received no treatment and no injection of CFA, Group 2 (arthritis control) received vehicle (5% DMSO + PBS) with CFA injected in the paw, Group 3 (positive control) received indomethacin (5 mg/kg) with CFA injected in the paw, Groups 4 and 5 received aqueous extract of *P. africanum* (200 and 400 mg/kg) with CFA injected in the paw, Groups 6 and 7 methanol extracts of *P. africanum* (200 and 400 mg/kg) with CFA injected in the paw. All treatments was administered orally thirty minutes before CFA induction (day 0), then the animals were treated daily for up to 19th days.

#### Measurement of paw volume, joint diameter, arthritic score, pain threshold, thermal hyperalgesia and body weight

The severity of arthritis was evaluated on day 0, 1, 3, 5, 7, 9, 11, 13, 15, 17 and 19. For this purpose, Plethysmometer (UGO Basile, Italy) was used to measure paw volume [48]. Digital Vernier caliper (Mitutoyo, Japan) was used to measure joint diameter [49]. Randall Selitto analgesiometer (UGO Basile, Italy) was used to measure mechanical pain threshold [50] and plantar test apparatus (Ugo Basile, Comerio, Italy) was used to measure thermal hyperalgesia [51]. For each animal, variation of edema, joint diameter, paw withdrawal latency responses (pain threshold) and paw withdrawal latencies (thermal hyperalgesia) were expressed as % values relative to the pre-administration value (100%) [52]. Evaluation of the degree of arthritis was assessed daily by visual observation. A score of 0–4 helped distinguish the different disease stages with a maximum value of 8 for each rat. Scores was attributed according to the parameters such as edema, erythema, malformation and incapacity to use the limb [53]. Body weight was recorded with the aid of a balance.

#### Measurement of organs weight and Biochemical estimations

On day 20, after anesthesia (using anesthetic ether), cardiac puncture was used to draw blood and introduce into a tube containing EDTA as anticoagulant and into another tube without anticoagulant; then liver, kidney, thymus and spleen were removed delicately and weighed. Hematological parameters like erythrocytes (RBC) and leukocytes (WBC) counts, hemoglobin (Hb), Hematocrit and platelets (PLT) were determined in blood with anticoagulant by the usual standardized laboratory method [54]. Otherwise, blood without anticoagulant was centrifuged for 5 min (4900 rpm) and the serum was collected, then serum AST, ALT, ALP, total protein, C-reactive protein (CRP) and Rheumatoid factor (RF) levels was also quantified [55].

#### Histopathological analysis of ankle joints

After sacrifice, the animal knee joints were removed and preserved in 10% formalin + PBS. After fixation and decalcification, sample was cut into 45 μm pieces, then hematoxylin and eosin (H & E) for microscopic evaluation [56].

### Statistical analysis

Mean ± standard Error of Mean (SEM) was used to express the results of the study. For statistical analysis, multiple comparisons of data were carried out using two and one way analysis of variance (ANOVA), and then post test of Tukey was used for post hoc analysis. Significance was statistically acceptable at a level of *P < 0.05*. Software program GraphPad InStat was used for all data analysis.

## Results

### In vitro assay

#### Effect of *P. africanum* extracts on intracellular ROS production

To evaluate myeloperoxidase dependent effect of extracts, luminol was used as probe. On human whole blood phagocytes, results showed that, extracts of *P. africanum* (3.1–100 μg/ml) possess significant inhibitory activity on intracellular ROS production; with IC_50_ values of 10.04 μg/ml (aqueous extract) and 4.02 μg/ml (methanol extract) (Table 1).

**Table 1:** IC_50_ value of aqueous and methanol extracts of *Piptadeniastrum africanum* on human whole blood evaluated by luminol and/or lucigenin amplified chemiluminescence

	Oxidative burst (IC_50_ μg/ml)						T-cells proliferation (IC_50_ μg/ml)	Cytotoxicity on 3T3 Cells (IC_50_ μg/ml)
	Luminol and Zymosan			Lucigenin and PMA				
	WB	PMNs	MQ	WB	PMNs	MQ		
Aqueous extract	10.04 ± 0.31	8.68 ± 0.52	11.64 ± 0.29	8.59 ± 0.12	5.74 ± 0.38	11.85 ± 0.36	29.30 ± 0.36	78.60 ± 0.35
Methanol extract	4.02 ± 0.10	2.29 ± 0.10	2.85 ± 0.12	4.89 ± 0.28	2.96 ± 0.07	2.80 ± 0.02	<1	6.13 ± 0.25
Ibuprofen	13.00 ± 0.30	12.97 ± 0.23	13.93 ± 0.52	13.73 ± 0.58	13.69 ± 0.61	14.30 ± 0.49	-	-
Prednisolone	-	-	-	-	-	-	<3.1	-
Cyclohexamide	-	-	-	-	-	-	-	0.1 ± 0.13

The IC_50_ values are presented as mean ± SD of triplicates. Where *WB* whole blood, *PMNs* polymorphonuclear leukocytes, *MQ* mice peritoneal macrophages

Concerning neutrophils, the extracts also showed potent inhibitory effect with an IC_50_ value of 2.29 μg/ml (methanol extract) and 8.68 μg/ml (aqueous extract) (Table 1); when tested on ROS produced from the mice peritoneal macrophages, methanol extract caused significant inhibitory activity (IC_50_ = 2.85 μg/ml), while aqueous extract shows an IC_50_ of 11.64 μg/ml (Table 1). Ibuprofen, used as reference product, showed an IC_50_ of 12.97 and 13.93 μg/ml, respectively for neutrophils and mice peritoneal macrophages.

#### Effect of *P. africanum* extracts on extracellular ROS production

Myeloperoxidase independent effect was studied using lucigenin as probe. The results showed that, on whole blood, neutrophils and macrophages, both methanol and aqueous extracts exhibited significant inhibition of ROS. The IC_50_ values observed were 4.89 and 8.59 μg/ml, respectively for methanol and aqueous extracts on whole blood, compared to the ibuprofen (IC_50_ = 13.73 μg/ml) (Table 1). When tested on neutrophils, extracts showed significant inhibitory activity with 2.96 and 5.74 μg/ml as IC_50_ respectively for methanol and aqueous extracts (Table 1). Similarly on mice macrophages methanol extract showed a potential inhibitory effect (IC_50_ = 2.80μg/ml), followed by aqueous extract (IC_50_ = 11.85 μg/ml) compared to ibuprofen (IC_50_ = 14.30 μg/ml) (Table 1).

#### Effect of *P. africanum* extracts on Production of Pro-Inflammatory Cytokines

**Fig. 1:**
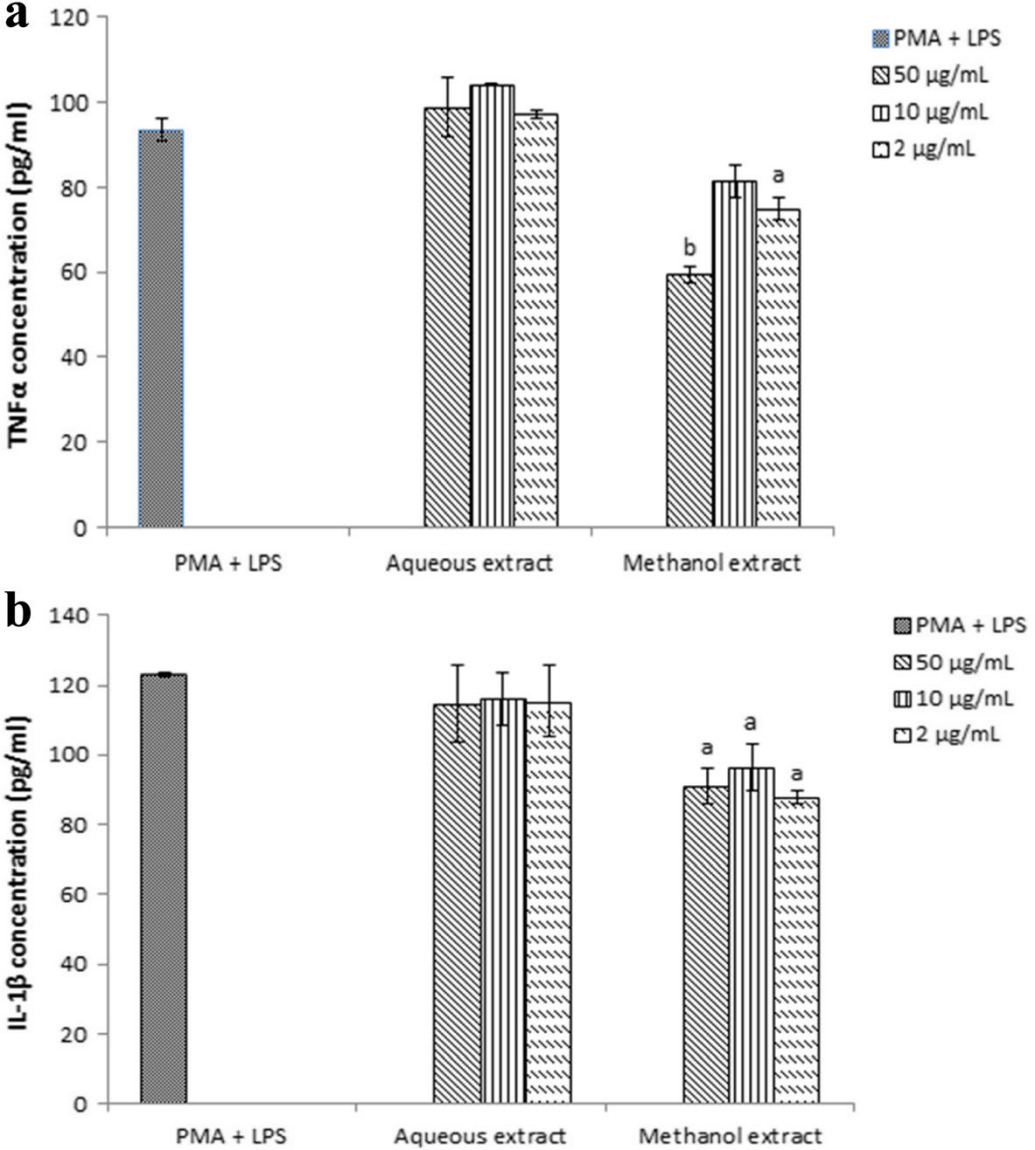
Effect of crude extracts of *Piptadeniastrum africanum* on TNF-α (**a**) and IL-1β (**b**) secretion levels by THP-1 activated by LPS. Data represent mean ± SD of triplicates. ^*a*^ *P < 0.05*, ^*b*^ *P < 0.01* when compared with that of the control: PMA + LPS

The effect of extracts on the release of TNF-α and IL-1β by activated THP-1 cells was evaluated with concentrations of 2–50 μg/ml of the extracts (Fig. 1). The methanol extract of *P. africanum* significantly (*P < 0.01*) decreased the level of TNF-α and IL-1β with 36.4% and 28.7% inhibitions respectively. On cytokines production, control samples and aqueous extract treated samples did not show any significant (*P > 0.05*) variations.

#### Effect of *P. africanum* extracts on T-Cell proliferation assay

On this assay, both extracts showed a notable level of antiproliferative effect, methanol extract caused the maximum inhibition with IC_50_ < 1 μg/ml, whereas aqueous extract showed a moderate level of antiproliferative effect with an IC_50_ = 29.30 μg/ml compared to prednisolone (IC_50_ < 3.1 μg/ml) (Table 1).

#### Cytotoxicity of the *P. africanum* extracts on 3T3 Cells

Aqueous and methanol extracts of *P. africanum* that showed potential inhibitory effects in various immunological assays were further tested for their possible toxicity effects. Aqueous extract was revealed to be devoid of toxic effect whereas methanol extract showed moderate level of toxicity on this cell line (IC_50_ = 6.13 μg/ml) compared to the cyclohexamide (IC_50_ = 0.1 μg/ml) used as the standard cytotoxic drug (Table 1).

### In vivo assay

#### Effect of *P. africanum* extracts on paw volume

**Fig. 2:**
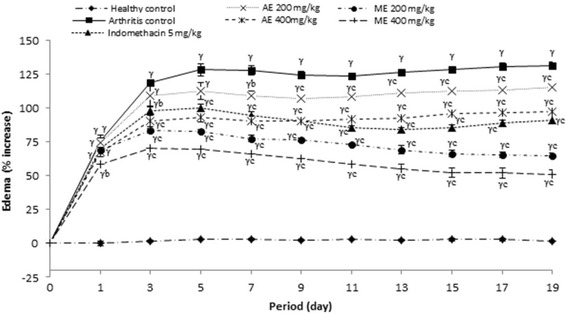
Effect of aqueous and methanol extracts of *Piptadeniastrum africanum* on Edema in FCA-induced arthritis. Values are expressed as mean ± SEM for six animals and analyses by two-way ANOVA followed by Tukey post-hoc test, ^*γ*^ *P < 0.001* when compared to healthy control, ^*b*^ *P < 0.01,* ^*c*^ *P < 0.001* when compared to arthritic control

Figure 2 reveals that, extracts of treated groups significantly (*P < 0.001*) lowered the paw volume from the first day (23.72% and 40.71% inhibition) until the end of treatment (25.92% and 61.10% inhibition) as compared to arthritis control group. Aqueous extract treated groups of 200 mg/kg was less effective, it significantly (*P < 0.01*) lowered edema from the 7th day till the last day of treatment. Indomethacin (5 mg/kg) significantly (*P < 0.001*) reduced the increase of edema induced by CFA from the 3rd day (17.38% inhibition) till the last day of treatment.

#### Effect of *P. africanum* extracts on joint diameter

**Fig. 3:**
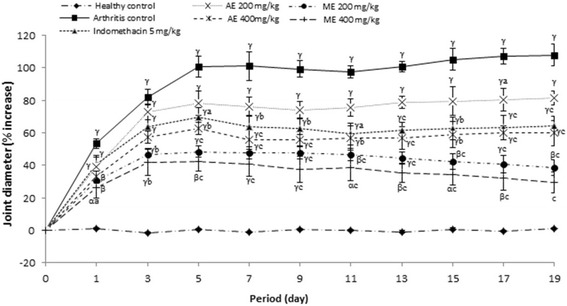
Effect of aqueous and methanol extracts of *Piptadeniastrum africanum* on change in joint diameter in CFA-induced arthritis. Values are expressed as mean ± SEM for six animals and analyses by two-way ANOVA followed by Tukey post-hoc test, ^*α*^ *P < 0.05,* ^*β*^ *P < 0.01,* ^*γ*^ *P < 0.001* when compared to healthy control, ^*a*^ *P < 0.05,* ^*b*^ *P < 0.01,* ^*c*^ *P < 0.001* when compared to arthritic control

Joint diameter of rats significantly (*P < 0.001*) increased in all rats on the groups treated with CFA. Methanol extract (400 mg/kg) considerably (*P < 0.001*) reduced the joint volume from the first day (43.03% and 49.10% inhibition) till the end of treatment (64.27% and 72.71% inhibition) as compared to arthritis control group. Aqueous extract or indomethacin exhibited an important (*P < 0.01; P < 0.05*) activity from the 5th day. Joint diameter variation was not significantly different among the methanol group (400 mg/kg) and healthy control on the 19th day after CFA injection (Fig. 3).

#### Effect of *P. africanum* extracts on mechanical nociceptive threshold

**Fig. 4:**
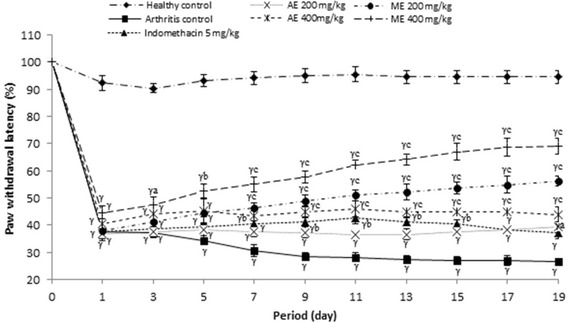
Effect of aqueous and methanol extracts of *Piptadeniastrum africanum* on mechanical nociceptive threshold in CFA-induced arthritis. Values are expressed as mean ± SEM for six animals and analyses by two-way ANOVA followed by Tukey post-hoc test, ^*γ*^ *P < 0.001* when compared to healthy control, ^*a*^ *P < 0.05,* ^*b*^ *P < 0.01,* ^*c*^ *P < 0.001* when compared to arthritic control

After the injection of CFA, the mechanical pain threshold decreased rapidly the first day and continued to decline gradually way until the 19th day as shown in the arthritic control. Significant protection against the mechanical pain was observed, from the first day (*P < 0.05*; 27.65% inhibition) until the end of the treatment for methanol extract (400 mg/kg, *P < 0.001*; 161.28% inhibition); from the 7th day (*P < 0.01*; 42.03% inhibition) until the end of the treatment for aqueous extract (*P < 0.001*; 66.18% inhibition) and methanol extract (*P < 0.001*). However, there was little improvement observed in aqueous extract (200 mg/kg) concerning mechanical withdrawal threshold. Indomethacin (5 mg/kg) showed significant improvement in mechanical withdrawal threshold between the 9th day (45.37% inhibition) and the 17th day (42.53% inhibition) only (Fig. 4).

#### Effect of *P. africanum* extracts on thermal hyperalgesia (paw withdrawal latency)

**Fig. 5:**
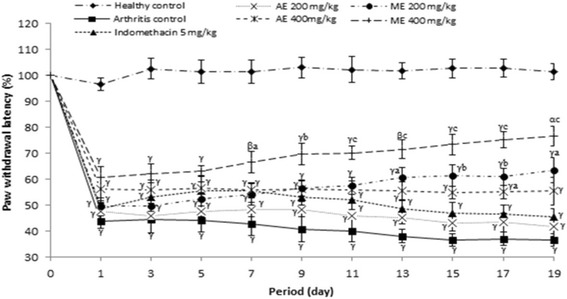
Effect of aqueous and methanol extracts of *Piptadeniastrum africanum* on thermal hyperalgesia in CFA-induced arthritis. Values are expressed as mean ± SEM for six animals and analyses by two-way ANOVA followed by Tukey post-hoc test, ^*α*^ *P < 0.05,* ^*β*^ *P < 0.01,* ^*γ*^ *P < 0.001* when compared to healthy control, ^*a*^ *P < 0.05,* ^*b*^ *P < 0.01,* ^*c*^ *P < 0.001* when compared to arthritic control

Figure 5 indicated the effect of extracts on paw withdrawal latency after injection of CFA on rat. On this figure it is observed that, methanol extract significantly (*P < 0.001*) increased the paw withdrawal latency from day 7 (55.87% inhibition) to day 19 (109.08% inhibition), while the same extract (200 mg/kg) was only significant from day 13 (P < 0.05; 59.34% inhibition). Indomethacin (5 mg/kg) as the aqueous extract (200 mg/kg) was not effective in this model.

#### Effect of *P. africanum* extracts on body weight

**Fig. 6:**
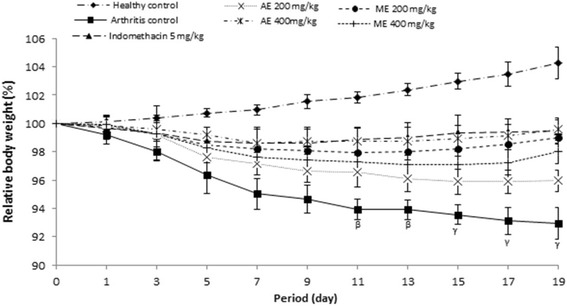
Effect of aqueous and methanol extracts of *Piptadeniastrum africanum* on body weight in CFA-induced arthritis. Values are expressed as mean ± SEM for six animals and analyses by two-way ANOVA followed by Tukey post-hoc test, ^*β*^ *P < 0.01,* ^*γ*^ *P < 0.001* when compared to healthy control

In untreated rats (arthritis control group), body weight decreased gradually and became significant (*P < 0.01*) from the 11th day compared to the animals of healthy group. In animals of different groups treated with extracts (400 and 200 mg/kg) as has indomethacin (5 mg/kg), change in body weight was not significant (*P > 0.05*) throughout the treatment compared to the animals of healthy groups (Fig. 6).

#### Effect of *P. africanum* extracts on arthritic score

**Fig. 7:**
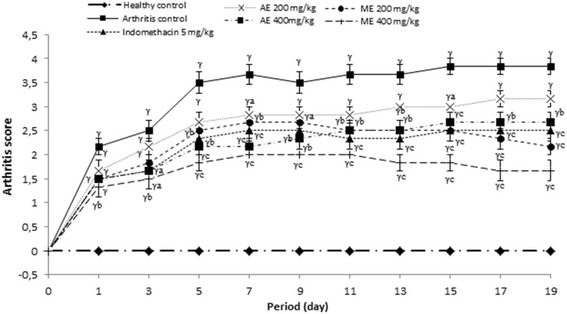
Effect of aqueous and methanol extracts of *Piptadeniastrum africanum* on arthritis score in CFA-induced arthritis. Values are expressed as mean ± SEM for six animals and analyses by two-way ANOVA followed by Tukey post-hoc test, ^*γ*^ *P < 0.001* when compared to healthy control, ^*a*^ *P < 0.05,* ^*b*^ *P < 0.01,* ^*c*^ *P < 0.001* when compared to arthritic control

Morphological variation materialized by the arthritic score was significant (*P < 0.001*) in all animals that received a sub-plantar administration of CFA. Aqueous and methanol extracts or indomethacin effectively protected the animals against the exaggeration of morphological variation observed in untreated animals; this was reflected by a significant variation (*P < 0.001*) of arthritic scores between animal treated groups and those of the untreated group (Fig. 7).

#### Effect of *P. africanum* extracts on hematological parameters

Table 2 shows the effects of the extracts on changes in hematological parameters 19 days after administration of the CFA. The results of this table reveal that, in animals of the untreated group, the levels of platelets and WBC are significantly increased (*P < 0.001*) while the levels of RBC, Hb and hematocrit are significantly decreased compared to animals of healthy group. Moreover, the results also show that, both extracts or indomethacin significantly attenuated these changes in such a way that, at 400 mg/kg, there is no significant variation between animals of the healthy group and those of the groups treated with aqueous and methanol extracts.

**Table 2:** Influence of the aqueous and methanol extracts of *Piptadeniastrum africanum* on haematological in CFA-induced arthritis in rats

	Dose (mg/kg)	Haemoglobin (g/dl)	RBC (million/μl)	Hematocrit (%)	WBC (10^9^/L)	Platelet (10^9^/L)
Healthy control	-	14.35 ± 0.45	7.37 ± 0.10	40.80 ± 2.50	7.45 ± 0.45	813.00 ± 40.00
Arthritic control	-	8.50 ± 0.20^γ^	3.99 ± 0.44^β^	26.75 ± 1.55^α^	13.25 ± 0.35^γ^	1815.00 ± 20.00^γ^
Indomethacin	5	11.60 ± 0.60^a^	6.05 ± 0.45	35.10 ± 2.20	10.00 ± 0.10^b^	1074.00 ± 26.00^αc^
Aqueous extract	200	12.05 ± 0.15^a^	5.90 ± 0.07	34.60 ± 0.90	8.80 ± 0.80^b^	1110.00 ± 21.00^αc^
	400	12.15 ± 0.65^a^	6.53 ± 0.13^a^	36.85 ± 1.05^a^	8.40 ± 0.30^b^	1043.50 ± 4.50^c^
Methanol extract	200	12.15 ± 0.35^a^	6.13 ± 0.57	35.00 ± 3.00	10.85 ± 0.85^α^	1134.00 ± 45.00^βc^
	400	12.45 ± 0.45^a^	6.89 ± 0.33^a^	38.60 ± 2.00^a^	8.85 ± 0.15^b^	919.00 ± 84.00^c^

*CFA* complete Freund’s adjuvant, *RBC* red blood cell, *WBC* white blood cell, *Hb* haemoglobin, *MCV* mean corpuscular volume, *MCH* mean corpuscular hemoglobin, *MCHC* mean corpuscular hemoglobin concentration. Each value represents the mean ± ESM of six animals. ^*α*^ *P < 0.05;* ^*β*^ *P < 0.01;* ^*γ*^ *P < 0.001* statistically significant compared to Healthy control. Each value represents the mean ± ESM of 6 animals*.* ^*a*^ *P < 0.05;* ^*b*^ *P < 0.01;* ^*c*^ *P < 0.001* statistically significant compared to Arthritic control

#### Effect of *P. africanum* extracts on various organ weights

The results showed that, liver, spleen and kidney weights increased considerably (*P < 0.01*) and thymus weight decreased significantly (*P < 0.01*) in all animals of the untreated group. Then, no significant change was observed between the organ weight of animals treated with extracts or indomethacin and organ weight of those of healthy groups (Table 3).

**Table 3:** Influence of the aqueous and methanol extracts of *Piptadeniastrum africanum* on organs weight after CFA induced arthritis

	Dose (mg/kg)	Liver	Spleen	Kidney	Thymus
Healthy control	-	6.69 ± 0.16	0.60 ± 0.01	0.91 ± 0.03	0.63 ± 0.04
Arthritic control	-	9.19 ± 0.29^β^	1.27 ± 0.04^β^	1.35 ± 0.08^β^	0.31 ± 0.01^β^
Indomethacin	5	7.45 ± 0.40^a^	0.81 ± 0.01^a^	0.98 ± 0.02^a^	0.54 ± 0.01^a^
Aqueous extract	200	7.85 ± 0.03	0.90 ± 0.05	1.12 ± 0.01	0.45 ± 0.04
	400	6.61 ± 0.09^b^	0.77 ± 0.05^a^	0.91 ± 0.09^b^	0.54 ± 0.02^a^
Methanol extract	200	7.55 ± 0.30^a^	0.89 ± 0.01	1.03 ± 0.04^a^	0.59 ± 0.06^b^
	400	6.55 ± 0.30^c^	0.74 ± 0.14^b^	0.93 ± 0.06^b^	0.62 ± 0.01^b^

Each value represents the mean ± ESM for six animals and analyses by two-way ANOVA followed by Tukey post-hoc test, ^*β*^ *P < 0.01* when compared to healthy control, ^*a*^ *P < 0.05,* ^*b*^ *P < 0.01,* ^*c*^ *P < 0.001* when compared to arthritic control

#### Effect of *P. africanum* extracts on Biochemical parameters

The results in Table 4 show that, in untreated animals (arthritic control group), serum levels of CRP, RF, AST, ALT and ALP significantly increased (*P < 0.001*) and total protein level significantly decreased (*P < 0.001*) compared to the parameters of animals of the healthy group. In animals treated with extracts or indomethacin, all biochemical parameters evaluated tend to return to normal values.

**Table 4:** Effect of aqueous and methanol extracts of *Piptadeniastrum africanum* on serum parameters in CFA-induced arthritis in rats

	Dose (mg/kg)	CRP (mg/l)	RF (IU/ml)	ALP (U/l)	ALT (U/l)	AST (U/l)	Total protein (g/dl)
Healthy control	-	1.69 ± 0.01	-	71.67 ± 2.73	44.73 ± 2.60	41.33 ± 1.86	6.69 ± 0.58
Arthritic control	-	6.92 ± 0.24^γ^	58.00 ± 1.53	471.00 ± 13.23	186.53 ± 11.93^γ^	132.67 ± 4.10^γ^	4.83 ± 0.12^β^
Indomethacin	5	3.84 ± 0.30^γc^	40.33 ± 1.86^c^	204.33 ± 46.04^βc^	123.29 ± 4.41^γc^	101.67 ± 1.76^γc^	5.85 ± 0.45^α^
Aqueous extract	200	6.11 ± 0.32^γ^	51.67 ± 1.76	416.33 ± 14.33^γ^	153.76 ± 8.69^γa^	125.09 ± 2.65^γ^	5.01 ± 0.07
	400	3.98 ± 0.05^γc^	40.00 ± 1.54^c^	340.07 ± 26.15^γb^	121.67 ± 2.03^γc^	113.77 ± 7.88^γa^	5.38 ± 0.25
Methanol extract	200	4.29 ± 0.19^γc^	40.33 ± 0.88^c^	214.13 ± 4.67^γc^	119.19 ± 1.45^γc^	93.33 ± 3.48^γc^	5.53 ± 0.10
	400	3.04 ± 0.04^βc^	31.00 ± 1.16^c^	131.60 ± 5.24^c^	82.21 ± 6.66^αc^	74.56 ± 5.78^γc^	6.40 ± 0.26^a^

*CFA* complete Freund’s adjuvant, *CRP* C-reactive protein, *RF* rheumatoid factor, *ALP* alkaline phosphatase, *AST* aminotransferase, *ALT* alanine aminotransferase. Each value represents the mean ± ESM for six animals and analyses by two-way ANOVA followed by Tukey post-hoc test, ^*α*^ *P < 0.05*, ^*β*^ *P < 0.01*, ^*γ*^ *P < 0.001* when compared to healthy control, ^*a*^ *P < 0.05*, ^*b*^ *P < 0.01*, ^*c*^ *P < 0.001* when compared to arthritic control

#### Histology of ankle joints

**Fig. 8:**
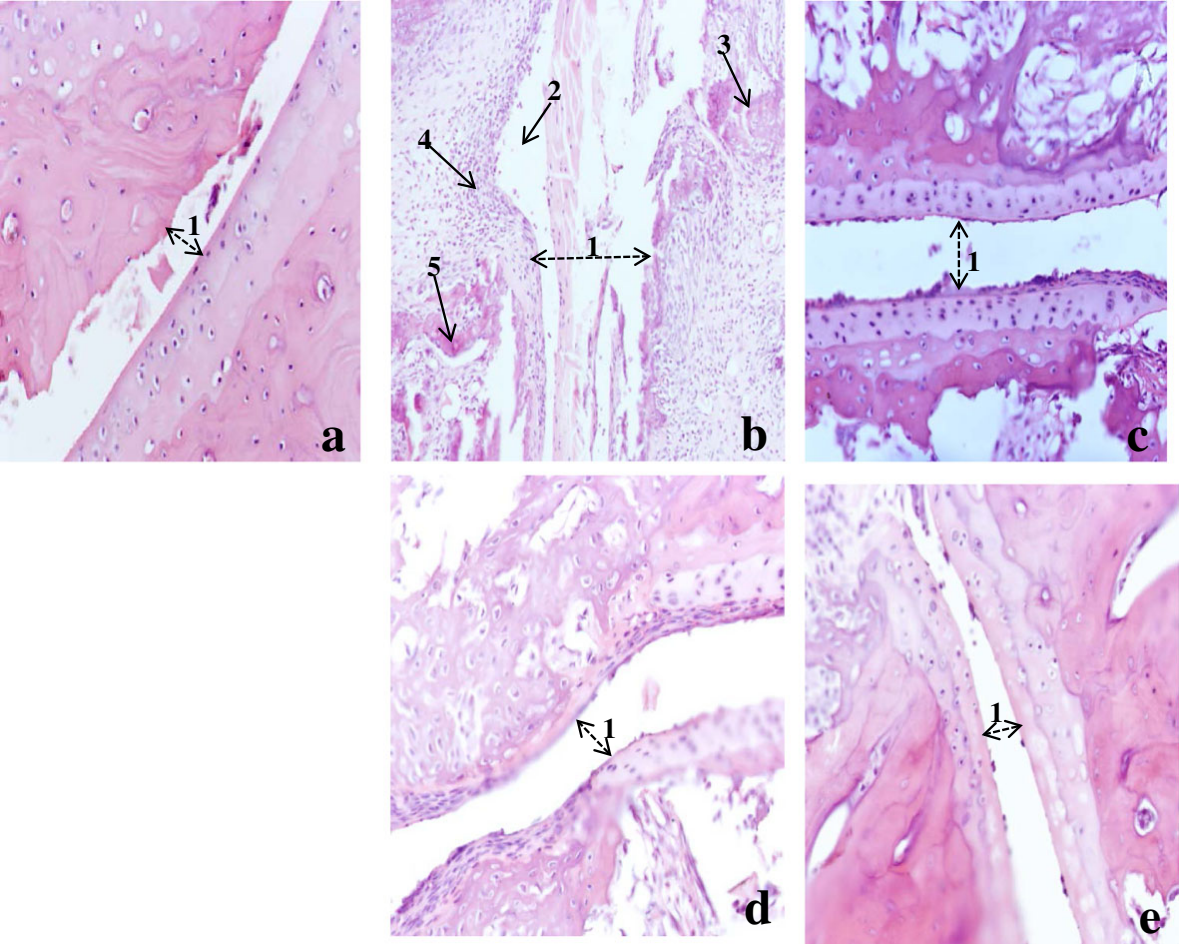
Histopathological analysis of ankle joints stained with H&E. **a** Healthy control shows normal structure with small joint space; **b** Arthritic control which shows very large joint space (1), severe hyperplasia (2), granulomas (3), cells infiltration (4) and erosion (5); indomethacin 5 mg/kg treated (**c**), aqueous extract 400 mg/kg treated (**d**) and methanol extract 400 mg/kg treated (**e**) show a decrease in joint space (1) and a reduction of cells infiltration

Histopathology of the ankle joint of healthy control rats revealed no inflammation, a few lymphocytes infiltration and no bone necrosis. A massive influx of inflammatory cells, cartilage destruction, proliferation of granulation tissue, lymphocytes infiltration and chronic inflammation was detected in arthritic control. In contrast to these pathological changes, animals having received aqueous and methanol extracts of *P. africanum* or indomethacin showed significant protection against necrosis of bones with low influx of inflammatory cells and minimal bone damage compared (Fig. 8).

## Discussion

Rheumatoid Arthritis, with symptoms such as swelling (joints), release of RF (autoantibody), deformity (bone destruction) and systemic change, is a more frequent disease that presents major systemic clinically complications with a high mortality rate in patients compared to healthy people [57]. In rheumatoid arthritis, swelling of the synovium due to the proliferation of synovial cells, is considered the main actor to affection and deterioration of cartilage with consequences such as the loss of the protective role of the synovial liquid by altering the binding properties of proteins in the cartilage [58]. Bone erosion, associated with increased and prolonged inflammation, affects 80% of patients and occurs rapidly [59, 60]. Osteoclast differentiation associated with incursion of cells in the periosteal observed at surface contiguous to articular cartilage are due to cytokines released in the synovium, principally [61]. In addition, differentiation and activation of osteoclasts are amplified by TNF-α and interleukin 1 and 6 [62]. Moreover, in clinical management, bone erosion can be delayed by inhibition of TNF-α, interleukin1 and RANKL [63]. With the capacity to activate cytokine and chemokine expression, TNF-α plays an important and decisive role on endothelial cell adhesion molecules, on angiogenesis, on suppression of regulatory T-cells, on protection of synovial fibroblasts and induction of pain [63, 64], while, cartilage lesions and delayed healing lesions are due to IL-1β [63].

The studies demonstrate that extracts of *P. africanum* inhibited considerably the release of TNF-α and IL-1β. The results suggest that, the inhibition induced by the methanol extract on cytokine production possesses clinical significance. Inhibition of release of TNF-α and IL-1β in presence methanol extract of *P. africanum* specifies that, this plant possesses compounds which have anti-inflammatory potential associated with the capacity to intervene in the immune response. Otherwise, inhibitor effect of new compounds on TNF-α and IL-1β release is a valid approach because of their major role in differentiation, growth and death of immune cells and in the treatment of several inflammatory diseases [65].

To study the effect of aqueous and methanol extracts of *P. africanum* another aspect of cellular immune response, T-cell proliferation assay was used. The results show that, these extracts significantly inhibited T-cell proliferation and dose response relationship was observed, with a significant result for the extracts versus the positive control (prednisolone) of cell proliferation. Given that, in the immune response, induction of the hepatic acute phase protein response and influence of T-cell responses are part of the assigned functions [66], it is possible that the inhibitory effect on cell proliferation was the result of decreased cytokine TNF-α and IL-1β production. The results suggest that, the compounds of *P. africanum* were capable to modulate significantly at different steps, the immune response of phagocytes and monocytes.

In pathogenicity of RA, ROS also plays a decisive role; the lack of control of ROS production improves the bones and cartilage destruction and activate or suppress NF-kB [40, 67]. *P. africanum* showed excellent antioxidant capacity in this study, by significantly inhibiting the ROS production on whole blood and various phagocytic cells. It is possible that, this effect might be associated with the action of the extracts on the NF-kB, given that the inhibition effect of many compounds on NF-kB phosphorylation considerably suppresses the proliferation of T-cells [67], but most probably it could be due to the effect of the presence of some triterpenoids compound; given that, in our previous work, several triterpenoids compound like betulinic acid and oleanic acid, were obtained from the methanol extract of *P. africanum* [68]. These triterpenoids possess anti-inflammatory and/or antiproliferative properties by the inhibitory effect of NF-kB phosphorylation [69, 70]. The NF-kB/AP1 axes is important for the inflammatory reaction with stimulation of the release of TNF-α, IL-1β, NO and PGE_2_ [71, 72], the suppression of this axe has a crucial therapeutic effect [73]. The clinical use of compounds capable of inhibiting the production of TNF-α and IL-1β confirmed their importance [74]. Moreover, extracts of *P. africanums* show the in vitro inhibition of TNF-α and IL-1β stimulated by PMA and LPS.

In the present study, the cytotoxic activity of different extracts of *P. africanum* was evaluated on 3T3 cell line. The aqueous extract showed no toxic effect (IC_50_ = 78.6 μg/ml) whereas the methanol extract showed a moderate toxicity (IC_50_ = 6.13 μg/ml). To discover the toxicity of a compound in humans, and to generally identify toxic products, in vitro cytotoxicity assays are generally used [75, 76]. Depending on the test agent used and the cytotoxicity assay employed, cytotoxicity tests can give different results [77]. Therefore, more than one assay should be necessary to determine cell viability in in vitro essay and this would increase the reliability of the results obtained. However, Assob et al. [35] showed that, administered in the acute treatment, the methanol extract of *P. africanum* as the aqueous extract causes no death in rats during acute toxicity showing an LD_50_ > 5 g/Kg *b.w.* which classifies this plant among the nontoxic plants [78, 79].

The experimental model of polyarthritis induced by CFA on rat is widely used for preclinical testing of numerous anti arthritic agents [80]. This model, due to its close similarities with human rheumatoid diseases, is widely used to evaluate inflammatory disease and valid as a chronic pain model [81, 82]. In the present study, extracts of *P. africanum* treatment showed antiarthritic potential in all the inflammatory parameters. It significantly decreased the inflammation in treated animals by reducing the paw volume, joint diameter and arthritic score. In addition, the significant decrease on body weight observed on animals in the arthritis group was completely corrected by the extracts. Weight loss is a powerful predictor of health especially in pathological states [83]. In the case of RA, weight loss would be due to muscle loss, poor appetite and metabolic burden of inflammatory response [84]. On these important parameters, the inhibitory effect of extracts was significantly higher than that of indomethacin. After injection of CFA, there occurs a modification in transduction sensitivity of high threshold nociceptors with as consequence the appearance of hyperalgesia and allodynia [85]. The analgesic effect of extracts of *P. africanum* in rats with adjuvant arthritis is also marked as evident by the significant decrease of hyperalgesia (thermal and mechanical).

In arthritic patients, serum CRP, prototype biomarker of systemic inflammation for acute phase reactants with a level that increases rapidly during inflammatory processes, is used as a useful serum biomarker for evaluating the active inflammation [86, 87]. Serum rheumatoid factor (RF) is an immunoglobulin molecule considered as "non self” capable of eliciting a reaction of the immune system [88]. In the pathogenesis of RA, abnormal changes in serum level of RF and CRP could be recognized as a strong indicator of RA [89]. CFA induced arthritis in rats increased the CRP and RF level as evidenced in the inflammatory process as shown by the results of the arthritis control group in this study. A significant (*P < 0.001*) decrease of RF and CRP level was observed after treatment with aqueous and methanol extracts of *P. africanum*.

To evaluate anti-arthritic activity of a drug, the level of AST ALT ALP and total protein provide an excellent and simple tool. Aminotransferases and ALP which are good indices of liver as kidney impairment, their activities significantly increased in adjuvant arthritis in rats [90]. In addition to the fact that the activity of serum alkaline phosphatase increased in pathogenicity of RA, the serum level of this enzyme like that of AST would play an important role in the release of biologically active compounds (bradykinins) in the inflammatory process [91, 92]. This enzyme being liberated into circulation during the bone formation and resorption, will be involved in localized bone loss as bone erosion and periarticular osteopenia [93]. In addition, in about 30% of patients with RA, a significant elevation of serum ALT levels was observed [94]. In this study, arthritic rats showed significantly higher values of serum ALP, AST and ALT, while in animals with the different treatments (*P. africanum* or indomethacin), increased levels of these enzymes was significantly attenuated.

Suppressive activity of the extracts on fundamental molecules (TNF-α and T-cells) which play an essential role in the pathogenesis of arthritis [92] observed in vitro could justify the net suppressive effect on inflammation and hyperalgesia. This effect could be at the origin of the observation of decreased levels of CRP and RF in serum, also in the maintenance of animal body weight, given that TNF-α plays a key role in the genesis of asthenia and weight loss in RA [11]. The decrease in edema and joint diameter observed macroscopically and histopathologically followed by increase in latency time of pain threshold and thermal hyperalgesia clearly reveals the anti-inflammatory, antihyperalgesia and anti-arthritis potential of *P. africanum*.

## Conclusion

After this study, we can say that *P. africanum* is a plant rich in compounds possessing anti-inflammatory, antihyperalgesic and/or anti-arthritic potential. These properties were evaluated by in vivo study using a model of CFA induced on rats. The activity of the extracts of this plant is strongly justified by its effect on the immune system and/or inhibitory properties to the release of pro-inflammatory mediators as observed in the in vitro study. These results justify the use of this plant for decades in traditional treatment against inflammatory diseases including arthritis and classify this plant among the potential candidates for the isolation of novel anti-inflammatory and/or anti-arthritic products.

## Abbreviations

AIA: Adjuvant-induced arthritis
ALP: Alkaline phosphatase
ALT: Alanine aminotransferase
ANOVA: One-way analysis of variance
AST: Aspartate aminotransferase
CFA: Complete Freund's adjuvant
COX: Cyclooxygenase
CRP: C-reactive protein
DMEM: Dulbecco's Modified Eagle's medium
DMSO: Dimethylsulfoxide
ECACC: European Collection of Cell Cultures
ELISA: Enzyme-linked immunosorbent assay
FBS: Fetal bovine serum
H & E: Hematoxylin and eosin
Hb: Haemoglobin
HBSS: Hanks Balance Salts Solution
HEPES: 4-(2-hydroxyethyl)-1-piperazineethanesulfonic acid
IL-: Interleukin
LPS: Lipopolysaccharide
LSM: Lymphocytes Separation Medium
MAPK: Mitogen-activated protein kinases
MTT: 3-(4,5-Dimethylthiazol-2-yl)-2,5-diphenyltetrazolium bromide
NF-κB: Nuclear factor-kappa B
PBMNCs: Peripheral blood mononuclear cell
PCMD: Dr. Panjwani Center for Molecular Medicine and Drug Research
PGE_2_: Prostaglandins E_2_
PHA: Phytohemagglutinin
PLT: Platelets
PMA: Phorbol 12-myristate 13-acetate
PMNs: Polymorpho neutrophils
RA: Rheumatoid arthritis
RANKL: Receptor activator of NF-κB ligand
RBC: Red blood cell
RF: Rheumatoid factor
RLU: Relative light units
ROS: Reactive oxygen species
RPMI: Roswell Park Memorial Institute medium
RT: Room temperature
SEM: Standard error of mean
TNF-α: Tumor necrosis factor-α
WBC: White blood cell

## References

[1] Gabriel SE (2001). Epidemiology of rheumatoid arthritis. Rheum Dis Clin North Am.

[2] Symmons DP, Barrett EM, Bankhead CR, Scott DG, Silman AJ (1994). The incidence of rheumatoid arthritis in the United Kingdom: results from the Norfolk Arthritis Register. Br J Rheumatol.

[3] NCCCC (National Collaborating Centre for Chronic Conditions) (2009). Rheumatoid arthritis: National clinical guideline for management and treatment in adults.

[4] Gibofsky A (2012). Overview of epidemiology, pathophysiology, and diagnosis of rheumatoid arthritis. Am J Manag Care.

[5] Butler SH, Godefroy F, Besson JM, Weil-Fugazza J (1992). A limited arthritic model for chronic pain studies in the rat. Pain.

[6] Wang Q, Kuang H, Su Y, Sun Y, Feng J, Guo R, Chan K (2013). Naturally derived antiinflammatory compounds from Chinese medicinal plants. J Ethnopharmacol.

[7] Escandell JM, Recio MC, Máñez S, Giner RM, Cerdá-Nicolás M, Ríos JL (2006). Dihydrocucurditacin B, isolated from cayaponiatayuya, reduces damage in adjuvant induced arthritis. Eur J Pharmacol.

[8] Whidente GT, Boulet JM, Walker K (2005). The role of central peripheral μ opioïd receptors ininflammatory pain and edema: a study using morphine and DiPOA. J Pharmacol ExpTher.

[9] Firestein GS, Xu WD, Townsend K, Broide D, Alvaro-Gracia J, Glasebrook A, Zvaifler NJ (1988). Cytokines inchronic inflammatory arthritis. I. Failure to detect T cell lymphokines (interleukin 2 and interleukin 3) and presence of macrophage colony stimulating factor (CSF-1) and a novel mast cell growth factor in rheumatoid synovitis. J Exp Med.

[10] Firestein GS, Zvaifler NJ (2002). How important are T cells in chronic rheumatoid synovitis?: II. T cell-independent mechanisms from beginning to end. Arthritis Rheum.

[11] Bannwarth B, Bertin P, Binard A, Calvino B, Grilo RM, Saraux A, Sibilia J, Trèves R, Vergne-Salle P. Pain, inflammation and interaction nervous system/immune system. A Éditorial Paris, Institut UPSA de la douleur 2007; 135 pages.

[12] Holmberg J. Reduced ROS production triggers arthritis/The role of T cells in arthritis pathogenesis Medical Inflammation Research, Department of Experimental Medicine, Faculty of Medicine, Lund University 2004; 44pages.

[13] Hamdi H, Mariette X, Godot V, Weldingh K, Hamid AM, Prejean MV, Baron G, Lemann M, Puechal X, Breban M, Berenbaum F, Delchier JC, Flipo RM, Dautzenberg B, Salmon D, Humbert M, Emilie D (2006). the RATIO (Recherche sur Anti-TNF et Infections Opportunistes) Study Group. Inhibition of anti-tuberculosis T-lymphocyte function with tumour necrosisfactor antagonists. Arthritis Res Ther.

[14] Sibilia J, Sordet C (2005). Le rituximab : une biothérapie originale dans les maladies autoimmunes. Rev Med Interne.

[15] Feldmann M, Brennan FM, Maini RN (1996). Role of cytokines in rheumatoid arthritis. Annu Rev Immunol.

[16] Fairburn K, Stevens CR, Winyard PG, Kus M, Ward RJ, Cunningham J (1993). Oxidative stress and its control:apathogenetic role in inflammatory joint disease. Biochem Soc Trans.

[17] Tak PP, Zvaifler NJ, Green DR, Firestein GS (2000). Rheumatoid arthritis and p53: how oxidative stress might alter the course of inflammatory diseases. Immunol Today.

[18] Bauerova K, Bezek S. Role of Reactive Oxygen and Nitrogen Species in Etiopathogenesis of Rheumatoid Arthritis. Gen Physiol Biophys 1999; 18 Spec No: 15–20. 10703714

[19] Pandey S (2010). Various techniques for the evaluation of anti arthritic activity in animal models. J Adv Pharm Technol Res.

[20] Bendele A (2001). Animal models of rheumatoid arthritis. J Musculoskelet Neuronal Interact.

[21] Liu YL, Lin HM, Zou R, Wu JC, Han R, Raymond LN, Reid PF, Qin ZH (2009). Suppression of complete Freund's adjuvant-induced adjuvant arthritis by cobratoxin. Acta Pharmacol Sin.

[22] Geetha T, Varalakshmi P (1999). Anticomplement activity of triterpenes from Crataeva nurvala stem bark in adjuvant arthritis in rats. Gen Pharmacol.

[23] Andersen ML, Santos EHR, Seabra MLV, Da Silva AAB, Tufik S (2004). Evaluation of acute and chronic treatments with *Harpagophytum procumbens* on Freund’s adjuvant-induced arthritis in rats. J Ethnopharmacol.

[24] Davis L, Kuttan G (2000). Immunomodulatory activity of *Withania somnifera*. J Ethnopharmacol.

[25] Bin-Hafeez B, Haque R, Parvez S, Pandey S, Sayeed I, Raisuddin S (2003). Immunomodulatory effects of fenugreek (*trigonella foenum graecum l.*) extract in mice. Int Immunopharmacol.

[26] Cheng JLW, You T, Hu C (2005). Anti-inflammatory and immunomodulatory activities of the extracts from the inflorescence of *Chrysanthemum indicum* Linné. J Ethnopharmacol.

[27] Jadhav H, Singh A, Bhutani K (2005). Rationale for immunomodulatory and anti-inammatory effects of *Ocimum sanctum*: radical scavenging potential and effect on nitric oxide production. Acta Hort.

[28] Jiofack RB, Louppe D, Oteng-Amoako AA, Brink M (2008). *Piptadeniastrum africanum* (Hook). Brenan. Prota 7 (1): Timbers/Bois d'oeuvre 1.

[29] Noumi E, Yomi A (2001). Medicinal plants used for intestinal diseases in Mbalmayo Region, Central Province, Cameroon. Fitoterapia.

[30] Betti JL (2002). Medicinal plants sold in Yaounde markets. Cameroon Afr Study Monogr.

[31] Sumitra S, Nidhi S (2016). Evaluation of in vitro anti arthritic activity of *Acacia auriculiformis* A. Cunn. Ex. Benth. stem bark. World J Pharm Pharm Sci.

[32] Samrat C, Lalit K, Navpreet K, Randhir S (2015). Potential Anti-Arthritic Agents From Indian Medicinal Plants. Research and Reviews. J Pharm Pharm Sci.

[33] Pathak N, Gohil P, Patel NB, Kasture S, Jivani N, Bhalodia Y (2009). Curative Effect of *Albizia lebbeck* Methanolic Extract against Adjuvant Arthritis-With Special Reference to Bone Erosion. Int J Pharm Sci Drug Res.

[34] Mengome LE, Feuya TGR, Eba F, Nsi-Emvo E (2009). Antiproliferative Effect of Alcoholic Extracts of Some Gabonese Medicinal Plants on Human Colonic Cancer Cells. Afr J Tradit Complement Altern Med.

[35] Assob JCN, Kamga HLF, Nsagha DS, Njunda AL, Nde PF, Asongalem EA, Njouendou AJ, Sandjon B, Penlap VB (2011). Antimicrobial and toxicological activities of five medicinal plant species from Cameroon Traditional Medicine. BMC Complement Altern Med.

[36] Ateufack G, Domgnim MCE, Mbiantcha M, Dongmo FBR, Nana D, Kamanyi A (2015). Gastroprotective and ulcer healing effects of *piptadeniastrum Africanum* on experimentally induced gastric ulcers in rats. BMC Complement Altern Med.

[37] Diffoum JB. Propriétés analgésiques et anti-inflammatoires de l’extrait aqueux des écorces de Piptadeniastrum africanum (Mimosaceae) chez le rat. LAPHYPHA, UDs: *Thèse de MASTER* 2012; 94pages

[38] Note OP, Tapondjou AL, Mitaine-offer AC (2013). iyamoto TM, Pegnyemb DE, Lacaille-Dubois MA. Triterpenoid saponins from Piptadeniastrum africanum (Hook. f.) Brenan. Phytochem Lett.

[39] Chandrasenan P, Neethu MV, Anjumol VM, Anandan V, Selvaraj R (2016). Triterpenoid fraction isolated from *Euphorbia tirucalli Linn.* ameliorates collagen induced arthritis in Wistar rats. J App Pharm Sci.

[40] Almas J, Mesaik MA, Shabana US, Lubna SB, Shaheen F (2016). Anti-TNF-α and anti-arthritis of patuletin: A rare flavonoid from *Tagetes patula*. Int Immunopharmacol.

[41] Rita C, Bruno V, Helena R, Ana NC, Nuno F, Vineet G, João EF, Luis FM. Potent Anti-Inflammatory and Antiproliferative Effects of Gambogic Acid in a Rat Model of Antigen-Induced Arthritis. Mediators of Inflammation 2014; 7pages 10.1155/2014/195327 PMC3929289 24623960

[42] Zimmerman M (1983). Ethical guidelines for investigations of experimental pain in conscious animals. Pain.

[43] Mesaik MA, Ul-Haq Z, Murad S, Ismail Z, Abdullah NR, Gill HK, Yousaf M, Siddiqui RA, Ahmad A, Choudhary MI (2006). Biological and molecular docking studies on coagulin-H: Human IL-2 novel natural inhibitor. Mol Immunol.

[44] Mahomoodally MF, Gurib-Fakim A, Subratty AH (2007). Effect of exogenous ATP on Momordica charantia Linn. (Cucurbitaceae) induced inhibition of D-glucose, L-tyrosine and fluid transport across rat everted intestinal sacs in vitro. J Ethnopharmacol.

[45] Singh U, James T, Senthil KV, Sridevi D, Ishwarlal J (2005). Development of an In Vitro Screening Assay to Test the Anti-inflammatory Properties of Dietary Supplements and Pharmacologic Agents. Clin Chem.

[46] Scholz G, Pohl I, Genschow E, Klemm M, Spielmann H (1999). Embryotoxicity Screening Using Embryonic Stem Cells in vitro: Correlation to in vivo Teratogenicity. Cells Tissues Organs.

[47] Taniguchi N, Kanai S, Kawamoto M, Endo H, Higashino H (2004). Study on application of static magnetic field for adjuvant arthritis rats. Evid Based Complement Altern Med.

[48] Winter CA, Risley EA, Nuss CW (1962). Carageenan induced edema in hind paw of the rats as an assay for anti-inflammatory drugs. Proc Soc Exp Biol Med.

[49] Bihani GV, Rojatkar SR, Bodhankar SL (2014). Anti-arthritic activity of methanol extract of Cyathocline purpurea (whole plant) in Freund’s complete adjuvant-induced arthritis in rats. Biomedicine & Aging Pathology.

[50] Randall LO, Selitto JJ (1957). A method for measurement of analgesic activity on inflamed tissue. Arch Int Pharmacodyn Ther.

[51] Hargreaves K, Dubner R, Brown F, Flores C, Joris J (1988). A new and sensitive method for measuring thermal nociception in cutaneous hyperalgesia. Pain.

[52] Gabriella C, Maristella A, Elena G, Iwan JP (2007). de Esch, Rob L. Antiinflammatory and antinociceptive effects of the selective histamine H4-receptor antagonists JNJ7777120 and VUF6002 in a rat model of carrageenan-induced acute inflammation. Eur J Pharmacol.

[53] Foyet HS, Tsala DE, Zogo Essono Bodo JC, Carine AN, Heroyne LT, Oben EK (2015). Anti-inflammatory and antiarthritic activity of a methanol extract from *Vitellaria paradoxa* stem bark. Phcog Res.

[54] Mythilypriya R, Shanthi P, Sachdanandam P (2008). Salubrious effect of Kalpaamruthaa, a modified indigenous preparation in adjuvant-induced arthritis in rats – A biochemical approach. Chem Biol Interact.

[55] Mehta A, Sethiya N, Mehta C, Shah G (2012). Anti-arthritis activity of roots of *Hemidesmus indicus* R. Br. (Anantmul) in rats. Asian Pac J Trop Med.

[56] Patil K, Suryavanshi J (2007). Effect of Celastrus paniculatus Willd. seed on adjuvant induced arthritis in rats. Pharmacogn Mag.

[57] McInnes IB, Schett G (2011). The Pathogenesis of Rheumatoid Arthritis. N Engl J Med.

[58] Rhee DK, Marcelino J, Baker M (2005). The secreted glycoprotein lubricin protects cartilage surfaces and inhibits synovial cell overgrowth. J Clin Invest.

[59] van der Heijde DM (1995). Joint erosions and patients with early rheumatoid arthritis. Br J Rheumatol.

[60] Visser H, le Cessie S, Vos K, Breedveld FC, Hazes JM (2002). How to diagnose rheumatoid arthritis early: a prediction model for persistent (erosive) arthritis. Arthritis Rheum.

[61] Gravallese EM, Harada Y, Wang JT, Gorn AH, Thornhill TS, Goldring SR (1998). Identification of cell types responsible for bone resorption in rheumatoid arthritis and juvenile rheumatoid arthritis. Am J Pathol.

[62] Schett G, Teitelbaum SL (2009). Osteoclasts and arthritis. J Bone Miner Res.

[63] Feldmann M, Brennan FM, Maini RN (1996). Rheumatoid arthritis. Cell.

[64] Hess A, Axmann R, Rech J, Finzel S, Heindl C, Kreitz S, Sergeeva M, Saake M, Garcia M, Kollias G, Straub RH, Sporns O, Doerfler A, Brune K, Schett G (2011). Blockade of TNF-α rapidly inhibits pain responses in the central nervous system. Proc Natl Acad Sci U S A.

[65] Calamia KT (2003). Current and future use of anti-TNF agents in the treatment of autoimmune, inflammatory disorders. Adv Exp Med Biol.

[66] Akira S, Hirano T, Taga T, Kishimoto T (1990). Biology of multifunctional cytokines: IL-6 and related molecules (IL 1 and TNF). FASEB J.

[67] Almas J. Study of the suppression of inflammatory arthritis at molecular level by natural and synthetic inhibition of TNFα and IL-1β. Ph. D thesis 2013; 190 pages

[68] Dawé A, Mbiantcha M, Fongang Y, Nana WY, Yakai F, Ateufack G, Shaiq MA, Lubna I, Lateef M, Ngadjui BT. Piptadenin, a Novel 3,4-secooleanane Triterpene and Piptadenamide, a New Ceramide from the stem bark of *Piptadeniastrum africana (Hook. f.) Brenan*. Chem Biodiversity 2016; Accepted Author Manuscript. doi:10.1002/cbdv.201600215 10.1002/cbdv.201600215 27491939

[69] Rabi T, Shukla S, Gupta S (2008). Betulinic Acid Suppresses Constitutive and TNFα-induced NF-κB Activation and Induces Apoptosis in Human Prostate Carcinoma PC-3 Cells. Mol Carcinog.

[70] Yu-Jin H, Jaewhan S, Haeng-Ran K, Kyung-A H (2014). Oleanolic acid regulates NF-κB signaling by suppressing MafK expression in RAW 264.7 cells. BMB Rep.

[71] Silva JR L e, da Silva MD P, Lefort J, Vargaftig BB (2000). Endotoxins, asthma, and allergic immune responses. Toxicology.

[72] Harris SG, Padilla J, Koumas L, Ray D, Phipps RP (2002). Prostaglandins as modulators of immunity. Trends Immunol.

[73] Dudhgaonkar S, Thyagarajan A, Sliva D (2009). Suppression of the inflammatory response by triterpenes isolated from the mushroom *Ganoderma lucidum*. Int Immunopharmacol.

[74] Behm BW, Bickston SJ. Tumor necrosis factor-alpha antibody for maintenance of remission in Crohn's disease*.* Cochrane Database Syst Rev*.* 2008*,* Issue 1*.* Art. No.: CD006893. doi: 10.1002/14651858.CD006893*.* 10.1002/14651858.CD006893 18254120

[75] Clemedson C, Ekwall B (1999). Overview of the final MEIC results. I. The in vitro–in vitro evaluation. Toxicol In Vitro.

[76] Scheers ME, Ba E, Dierickx JP (2001). In vitro long-term cytotoxicity testing of 27 MEIC chemicals on HepG2 cells and comparison with acute human toxicity data. Toxicol In Vitro.

[77] Weyermann J, Lochmann D, Zimmer A (2005). A practical note on the use of cytotoxicity assays. Int J Pharmaceut.

[78] Delongeas JL, Burnel D, Netter P, Grignon M, Mur JM, Royer R, Grignon G (1983). Toxicity and pharmacokinetics of zirconium oxychloride in mice and rats. J Pharmacol.

[79] Lu FC. General data, Evaluation Procedures, target organs and assessment of risk. Toxicology Masson Paris 1992; 361pages.

[80] Benslay DN, Bendele AM (1991). Development of a rapid screen for detecting and differentiating immunomodulatory vs anti-inflammatory compounds in rats. Agents Actions.

[81] Colpaert FC, Meert T, Witte P, Schmitt P (1982). Further evidence validating adjuvant arthritis as an experimental model of chronic pain in the rat. Life Sci.

[82] Singh S, Majumdar DK (1996). Effect of fixed oil of *Ocimum sanctum* against experimentally induced arthritis and joint edema in laboratory animals. Int J Pharmacogn.

[83] Higgins M, D’Agostino R, Kannel W, Cobb J (1993). Benefits and adverse effects of weight loss: observations from the Framingham study. Ann Intern Med.

[84] Munro R, Capell H (1997). Prevalence of low body mass in rheumatoid arthritis: association with the acute phase response. Ann Rheum Dis.

[85] Fang JF, Liang Y, Du JY, Fang JQ (2013). Transcutaneous electrical nerve stimulation attenuates CFA-induced hyperalgesia and inhibits spinal ERK1/2-COX-2 pathway activation in rats. BMC Complement Altern Med.

[86] Milovanoic M, Nilson E, Jaremo P (2004). Relationship between platelets and inflammatory markers in rheumatoid arthritis. Clin Chim Acta J.

[87] Pepys MB, Hirchfield GM (2003). C-reactive protein: a critical update. J Clin Invest.

[88] Yildirim K, Karatay S, Melikoglu MA, Gureser G, Ugur M, Senel K (2004). Associations between acute phase reactant levels and disease activity score (DAS28) in patient with rheumatoid arthritis. Ann Clin Lab Sci.

[89] Rainsford KD (1982). Adjuvant polyarthritis in rats. Is this a satisfactory model for screening anti-arthritic drugs?. Agents Actions.

[90] Glenn EM, Gray J, Kooyers W (1965). Chemical changes in adjuvant induced polyarthritis of rats. Am J Vet Res.

[91] Niino-Nanke Y, Akama H, Hara M, Kashiwazaki S (1998). Alkaline phasphatase (ALP) activity in rheumatoid arthritis-its clinical significance and synthesis of ALP in RA synovium. Ryumachi.

[92] Rehman Q, Lane NE (2001). Bone loss. Therapeutic approaches for preventing bone loss in inflammatory arthritis. Arthritis Res.

[93] Aida S (1993). Relation between Rheumatoid Arthritis and Alkaline Phosphatase Isoenzymes. Ann Rheum Dis.

[94] Harris ED (1990). Rheumatoid arthritis: pathophysiology and implications for therapy. N Engl J Med.

